# High abundances of the nuisance raphidophyte *Gonyostomum semen* in brown water lakes are associated with high concentrations of iron

**DOI:** 10.1038/s41598-018-31892-7

**Published:** 2018-09-07

**Authors:** Karen Lebret, Örjan Östman, Silke Langenheder, Stina Drakare, François Guillemette, Eva S. Lindström

**Affiliations:** 1 Department of Ecology and Genetics/Limnology, Uppsala University, Norbyvägen 18D, SE-752 36 Uppsala, Sweden; 2 Centre for Ecology and Evolution in Microbial model Systems - EEMiS, Department of Biology and Environmental Science, Linnæus University, SE-391 82 Kalmar, Sweden; 3 Department of Aquatic Resources, Swedish University of Agricultural Sciences, Skolgatan 6, SE-742 42 Öregrund, Sweden; 4 Department of Aquatic Sciences and Assessment, Swedish University of Agricultural Sciences - SLU, PO Box 7050, SE-750 07 Uppsala, Sweden; 5 Research Center on Watershed – Aquatic Ecosystem Interactions (RIVE), Department of Environmental Sciences, Université du Québec à Trois-Rivières, Québec, Canada

## Abstract

Algal blooms occur frequently in lakes and oceans and the causes and consequences of those are often studied. In this study, we focus on a less well known type of algal bloom by the freshwater raphidophyte *Gonyostomum semen*. This species’ abundance and occurrence is increasing, especially in brown water lakes, the most abundant lake type in the boreal zone. The aim of the study was to investigate which environmental factors are associated with *G*. *semen* by statistical evaluation of field data of 95 Swedish lakes over five years. Although we found *G*. *semen* to be associated with dark waters it was, contrary to our expectations, mainly high concentrations of iron, and only to a lesser extent high TOC (total organic carbon) concentrations, that were associated with blooms of *G*. *semen*. In addition, high phosphorus concentrations and low pH also appear to facilitate *G*. *semen* blooms. We suggest that browning of lakes caused by increased iron concentrations may decrease net heterotrophy by fostering heavy algal blooms, i.e. the opposite to commonly assumed effects of increased DOM (dissolved organic matter).

## Introduction

Although algal blooms are important threats to the ecosystems and ecosystem services, not all types of algal blooms have received the same attention, neither by the public nor by the scientific community. Blooms by the freshwater raphidophyte *Gonyostomum semen* have increased both in frequency and occurrence over time in Fenno-Scandinavia^1–4^. In fact, blooms of *G*. *semen* can constitute more than 95% of the biomass of phytoplankton in lakes^5,6^, leading to a dramatic change in community structure with consequences for ecosystem functioning and energy flux, for instance because they are poorly grazed by zooplankton^7,8^. Thus, *Gonyostomum semen* is considered to be an invasive species in Fenno-Scandinavia^1,2,5^, but has still received relatively little attention. A previous study has suggested that the phenotypic plasticity toward pH and light conditions of *G*. *semen* might be an advantage for this species to invade new lakes^9^, still, it remains unclear which parameters facilitate the formation of blooms. The increase in *G*. *semen* over time may to some degree be explained by higher water temperatures and longer growing seasons^2^. However, increased temperature cannot explain why the blooms occur in an increasing number of lakes within the same climate zone^2^. It has further been suggested that *G*. *semen* could be promoted by a darker water color (i.e. browning or brownification) associated partly to increased dissolved organic matter (DOM) concentrations in boreal lakes^2,5,10^. Previous experimental studies have, however, shown contrasting results regarding the ability of *G*. *semen* to directly use dissolved organic matter through mixotrophy as a source of nutrients^9,11^, thus, the benefits for *G*. *semen* of waters with high organic matter concentrations remain unclear.

Brown water lakes are common in the boreal zone^12^ and the water color of these lakes has also been found to increase over time^13–15^. Increased water color of lakes may partly be due to an increase in DOM concentrations caused by, for instance, a change in land use from agriculture to forestry^13^, changes in precipitation input^16^ or climate^17^. However, a rise in iron concentrations may also have contributed to the increases in color^18^. Regardless of the many factors associated with the browning of lakes, increased color cause shifts in underwater light conditions as well as in the supply of macro- and micronutrients, such as organic carbon and iron, which can ultimately lead to an increased abundance and distribution of *G*. *semen* in lakes. However, so far studies have focused on the correlation between *G*. *semen* and the DOM concentrations and light conditions of lakes^2,10^, but little work has been invested in the potential link to the composition of the DOM or to increases in essential micronutrients such as iron.

In this study, we hypothesized that abundance of *G*. *semen* is associated with high water color and that the composition of the DOM should be an important factor for their success. DOM is composed of dissolved organic molecules that vary in composition and quality (including complexity and bioavailability). The origin of the DOM, allochthonous or autochthonous, can for instance influence its bioavailability^19^, which might affect the mixotrophic activity of plankton such as *G*. *semen*. Bioavailable DOM can be an important source of carbon and nitrogen for mixotrophic and heterotrophic organisms, and stimulate their growth^20–22^. To test our hypothesis we used a publicly available database on lake biology (i.e. phytoplankton) and chemistry (i.e. water color, total organic carbon (TOC) concentration, macro-nutrients, micronutrients, pH, temperature) of 95 annually monitored lakes from the Swedish national lake monitoring (http://miljodata.slu.se/mvm/, last accessed on 9^th^ April 2018), and statistically evaluated which environmental variables were associated with higher abundance of *G*. *semen* over a five-year period. In addition, we analyzed the potential impact of DOM quality, rather than just quantity, on *G*. *semen* abundance using a fluorescence approach in 72 of the 95 monitored lakes for one of the studied years. The quality of the DOM was investigated using fluorescence excitation-emission matrices (EEMs) in order to determine whether the DOM was of allochthonous or autochthonous origins. This study complements previous investigations on the subject by including additional essential micro-nutrients and also parameters to define the quality of the DOM which might be of importance for the growth of *G*. *semen*. The results show, as expected, that high abundances of *G*. *semen* were associated with high water color. However, this association appeared to be linked to high concentrations of iron in those lakes rather than high concentrations of total organic carbon (TOC) or any other aspect of the composition of the DOM. In addition, high abundance of *G*. *semen* was linked to high concentration of phosphorus and low pH as shown in previous studies^2,10^.

## Material and Methods

In order to determine the environmental factors that potentially influence the abundance of *G*. *semen*, we used chemical and phytoplankton data from the national lake inventory program of Sweden^23^ available publically from the national data host (http://miljodata.slu.se/mvm/, last accessed on 5^th^ April 2018). We analyzed data collected between 2010 and 2014 from the epilimnion of 95 lakes used for studying long-term trends in deposition and climate as they are not impacted from major urban point sources. We focused the analyses on the August data, where we had the most complete dataset, and as it is a period when *G*. *semen* can be abundant and form blooms^5,6^. Phytoplankton was sampled from boat in August by taking a water sample from the epilimnion with a 2-m long Plexiglas tube sampler (diameter = 3 cm). In lakes with a surface area >1 km^2^ a single mid-lake site was used for sampling. In lakes with a surface area <1 km^2^, five random epilimnetic water samples were collected, and mixed to form a composite sample from which a subsample was taken for analysis. Samples for phytoplankton analyses were preserved with Lugol’s iodine solution (2 g potassium iodide and 1 g iodide in 100 mL distilled water) supplemented with acetic acid. *Gonyostomum semen* cell counts were used as a measure of abundance in each lake. Cell abundances of *G*. *semen* were estimated within the monitoring program framework from phytoplankton samples using an inverted light microscope according to the Utermöhl technique^24^. To characterize the physical and chemical environment of the lakes, the following parameters from the database were used: latitude (Xcoord), longitude (Ycoord), surface water temperature (Temp), pH, conductivity measured at 25 °C (Cond), calcium (Ca), magnesium (Mg), sodium (Na), potassium (K), alkalinity (Alk), sulphate (SO_4_), chloride (Cl), ammonium-nitrogen (NH_4_), total nitrogen (TotN), total phosphorus (TotP), water color measured as absorbance of 0.45 μm filtered water in a 5 cm cuvette (AbsF420), silicon (Si), turbidity (Turb), total organic carbon (TOC), iron (Fe), and manganese (Mn). The chemical parameters were from the sample taken at 0.5 m depth and measured according to international (ISO) or European (EN) standards (SS-EN ISO 5667-1:2007)^23^.

In 2014 additional water samples were collected during the monitoring sampling and the water from 72 of the 95 lakes was filtered through 0.22 μm supor filter within 1 day of sampling for visible and fluorescence spectra measurements in order to characterize DOM quality. Absorbance scans were performed between 230–600 nm using a 1-cm quartz cuvette. The specific UV absorbance of organic carbon (SUVA_254_) was then calculated by dividing the decadic absorption coefficient at 254 nm by the DOC (dissolved organic carbon) concentrations^25^. Higher SUVA_254_ values are indicative of a higher aromaticity of the DOM pool, which have been related to increased terrestrial influence in aquatic systems^26^. Here, TOC concentrations were used instead of DOC as only TOC data were available within the monitoring framework.

Fluorescence excitation-emission matrices (EEM) were measured using filtered water between 240–450 nm (5 nm increments; excitation) and 300–600 nm (2 nm increments; emission)^27^, and corrected for inner filter effects^28^, and instrument-specific biases^29^. Fluorescence intensity was further normalized to Raman units (R.U.) based on the Raman area of pure water^30^. All corrections were performed using the FDOMcorr toolbox for Matlab version 1.6^31^. The EEMs were then used to determine the following indices: the fluorescence index (FI)^32^, freshness index (FRESH)^33^ and humification index (HIX)^34^. FI is a parameter allowing to determine whether the organic matter is from terrestrial or microbial origin^35^, and can be informative for the bioavailability of the DOM. FRESH is an indicator of the contribution of recently produced DOM^35^. HIX is an indicator of humic substance content, with higher values indicating higher humic content^35^, and can be an indication of bioavailability as humic substances are considered to be less available to organisms.

The EEMs were further modeled with parallel factor analysis (PARAFAC) using the drEEM toolbox and procedure^36^. Six fluorescence components were identified by the analysis, two of which are associated to visible humic-like substances (C1 and C4; ex/em: <260–330/464 nm and 275–405/512 nm, respectively), one microbial humic-like peak (C2; ex/em: 305/400 nm), one UV humic-like component (C3; ex/em: 380/434 nm), one tyrosine-like component (C5; ex/em: 270/316 nm), and one tryptophan-like component (C6; ex/em: 290/340 nm). All components have been identified in previous studies according to the online spectral library OpenFluor (www.openfluor.org; last accessed on June 8^th^ 2018)^37^. The model was validated using a split-half validation routine, core consistency diagnostic and a visual inspection of the model’s residuals to insure that no systematic signal was present.

In order to analyze which physical and chemical parameters were correlated with the abundance of *G*. *semen*, we used for each year individually a partial least square (PLS) regression modeling approach. PLS is a robust method that can handle both a large number of variables as well as collinearity among variables^38^. In order to reduce the effect of high variance and outliers in some of the variables, the abundance of *G*. *semen* and the explanatory variables were log transformed as log_10_(x + 0.01), and then all variables were standardized as z-scores, z = (x_i_ − average(x))/standard deviation (x), for each year. PLS analysis was performed in Simca 14^39^ and the number of latent components was determined by “Autofit” that maximize the predictive power of the model according to the cross-validation (based on seven subsets). From the PLS, we extracted Variable Importance for the Projection (VIP)-scores and loadings plots. VIP-scores represent the relative importance of each variable in the correlation with the dependent variable (*G*. *semen* abundance) and scores above 1 were considered important. In the loading plots variables close to *G*. *semen* are positively correlated and variables distant from *G*. *semen* are negatively correlated^40^. For year 2014 where EEM data were available, only the 72 lakes with EEM measurements were included in the PLS model.

**Figure 1:**
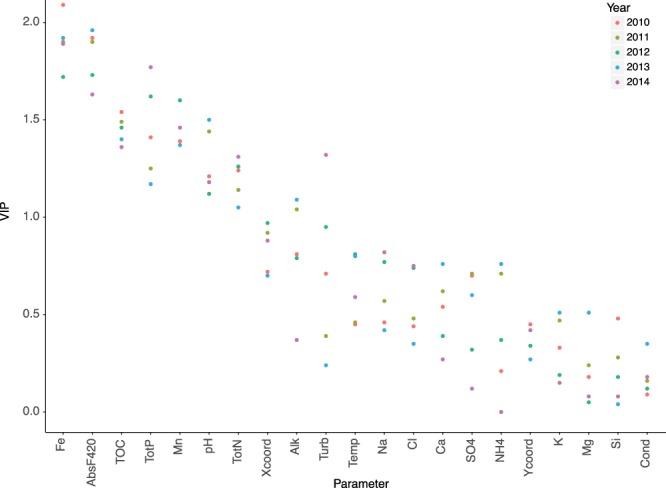
VIP values of the PLS analyses of the different explanatory variables for *G*. *semen* abundance for each year. Values above 1 are considered as important variables.

Based on the results from the PLS models we choose the explanatory variables with VIP > 1 across all years (Iron, AbsF420, pH, TOC, TotP, TotN, Table 1 and Fig. 1) as main predictive variables for further analyses. Mn was not included in the analysis due to the presence of several missing values. As predictive variables tend to be inter-correlated we used different methods to identify which variables were most important for *G*. *semen* abundance. First we investigated the marginal linear contribution of the respective predictive variable in stepwise regressions for each year with *G*. *semen* abundance as dependent variable. We used the function ‘*stepAIC’* in R 3.4.3 to select variables based on model AIC, and the function ‘*modelEffectSizes*’ to calculate partial r^2^-values.

**Table 1:** VIP scores of the PLS analysis of the 2014 data including the EEMs and PARAFRAC variables, in bold are the VIP values above 1 which are considered important factors.

Parameters	VIP
Fe	**2**.**18**
Absorbance 420 nm	**2**.**13**
Total phosphorus	**1**.**94**
TOC	**1**.**59**
Mn	**1**.**51**
Total Nitrogen	**1**.**48**
Turbidity	**1**.**43**
pH	**1**.**23**
HIX (Fluorescence index)	**1**.**10**
SUVA	0.90
%C5 (PARAFAC component)	0.89
FRESH (Fluorescence index)	0.80
Latitude	0.78
%C4 (PARAFAC component)	0.76
Na	0.74
%C6 (PARAFAC component)	0.70
Cl	0.62
%C1 (PARAFAC component)	0.57
%C3 (PARAFAC component)	0.52
Water Temperature	0.48
Alkalinity	0.37
Ca	0.35
Longitude	0.31
SO_4_	0.23
FI (Fluorescence index)	0.23
Si	0.15
K	0.14
NH_4_-N	0.09
Mg	0.09
Conductivity	0.07
%C2 (PARAFAC component)	0.01

To visualize the associations between the important predictive variables (with VIP > 1), excluding Mn due to the presence of missing values, and the abundance of *G*. *semen* across parameter space, partial dependence plots based on a Random Forest algorithm were made for each year using the package ‘randomForest’ for R 3.4.3^41^. The dependence plots show the contribution of one variable while controlling for the contribution of others. Partial dependence plots do not assume linear relationship and are useful to detect thresholds or unimodal relationships. The data were log-transformed prior to analyses with the exception of pH.

We also analyzed which explanatory variables differed statistically between different abundances of *G*. *semen* using regression tree models and the ‘rpart’ function in R 3.4.3^42^ for each year independently with the variables VIP > 1 identified in the PLS analyses. Regression trees are hierarchical models used to split up the dependent variable (abundance of *G*. *semen*) into one or several nodes of predicting explanatory variables. This means that predicting variables higher up are more important whereas variables further down have more context specific effects. Thus, regression trees show how different explanatory variables act in conjunction on the abundance of *G*. *semen* (note that a predictive variable can occur several time in a tree).

In addition, we decided to further investigate the importance of iron, pH and total phosphorus for the presence or absence of *G*. *semen* in the selected lakes. For this, the lakes were separated into two groups; lakes with occurrence of *G*. *semen* and the lakes with no *G*. *semen* during the 5 years period of 2010–2014. A non-parametric Wilcoxon test was used to determine if iron, total phosphorus concentrations and pH in these two groups were significantly different. A p-value < 0.05 was considered as significant.

## Results and Discussion

The aim of this study was to investigate possible reasons for bloom formation of *G*. *semen* in lakes. We focused here on lakes in Sweden where these blooms have become more frequent over time and hypothesized that this could be linked to the observed increase in water color in Swedish lakes and more specifically with the quantity and quality of DOM.

We first conducted a PLS analysis of data from 2014 including EEMs and PARAFAC analyses as a measure of DOM quality. The PLS analysis for 2014 with EEMs allowed to explained 38% of the variance in *G*. *semen* abundance (Supplementary Table S1). The results indeed showed that the abundance of *G*. *semen* was positively associated with water color (measured by the absorbance at 420 nm, Table 1 and Supplementary Fig. S1) in congruence with previous studies^2,10^, however, to a lesser extent to TOC. In addition, the fluorescence components extracted from the PARAFAC model to investigate the quality of the DOM were relatively unimportant parameters to explain the abundances of *G*. *semen*, according to the PLS analyses (Table 1). The abundance of *G*. *semen* was instead positively associated with high total phosphorus concentration, turbidity, Mn and total nitrogen concentrations, and lower pH (Table 1, and Supplementary Fig. S1). However, according to the VIP scores the concentration of iron was the strongest explanatory variable in the PLS analysis (Table 1). Therefore, we revised our initial hypothesis that the dependence of water color would be associated with TOC to also include that increased iron concentrations may lead to higher abundances of *G*. *semen*. In order to test this new hypothesis further we included data from four more years 2010–2013 of the same lakes. Since we did not have EEMs for these years, they were not included in further analysis.

The PLS analyses for 2010–2014 show high abundances of *G*. *semen* in association with dark water color within all years (Supplementary Fig. S1). The association with water color was, however, more closely related to iron concentrations than to TOC. In fact, according to VIP values, iron concentration was the strongest factor associated with *G*. *semen* abundance for most of the years, very close to water color (AbsF420), while TOC appeared to be of lower importance (Fig. 1). This indicates that the effect of water color on abundance of *G*. *semen* was to a greater degree explained by high iron concentration rather than TOC. Increase iron concentration has been shown in a previous study^18^ to be an important factor leading to a rise in water color and thus browning of freshwaters.

**Figure 2:**
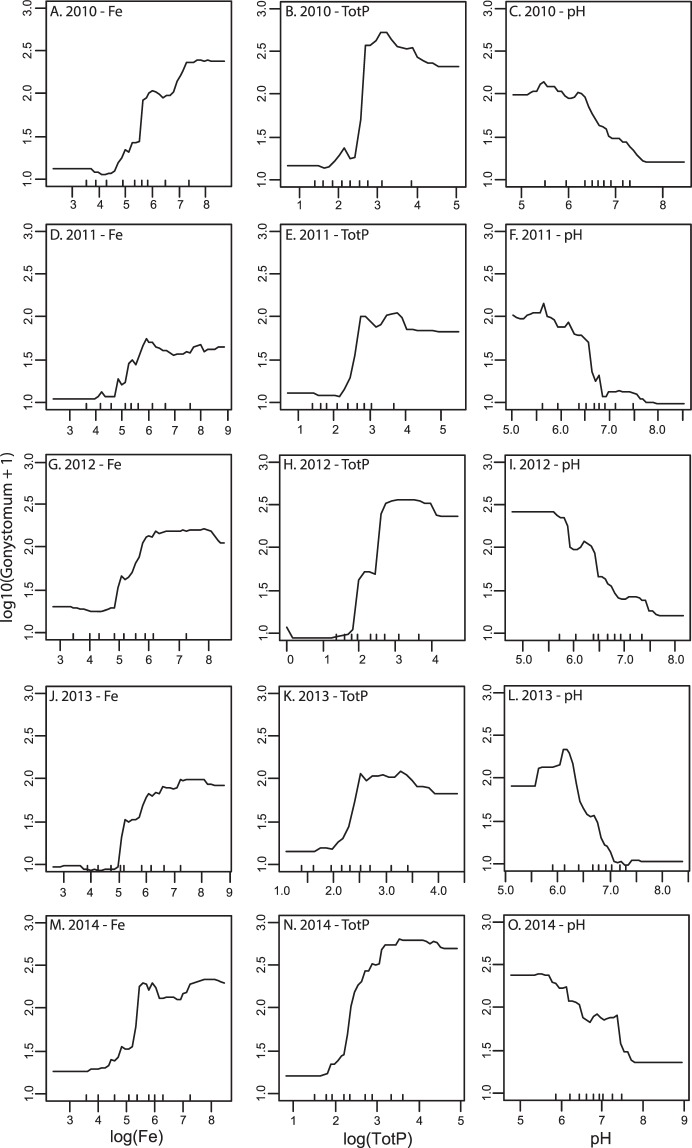
Partial dependency plots between *G*. *semen* abundances and iron concentrations (**A**,**D**,**G**,**J** and **M**) total phosphorus concentration (**B**,**E**,**H**,**K** and **N**) and pH (**C**,**F**,**I**,**L** and **O**) for 2010 to 2014. A linear relationship would show up as a straight line.

**Figure 3:**
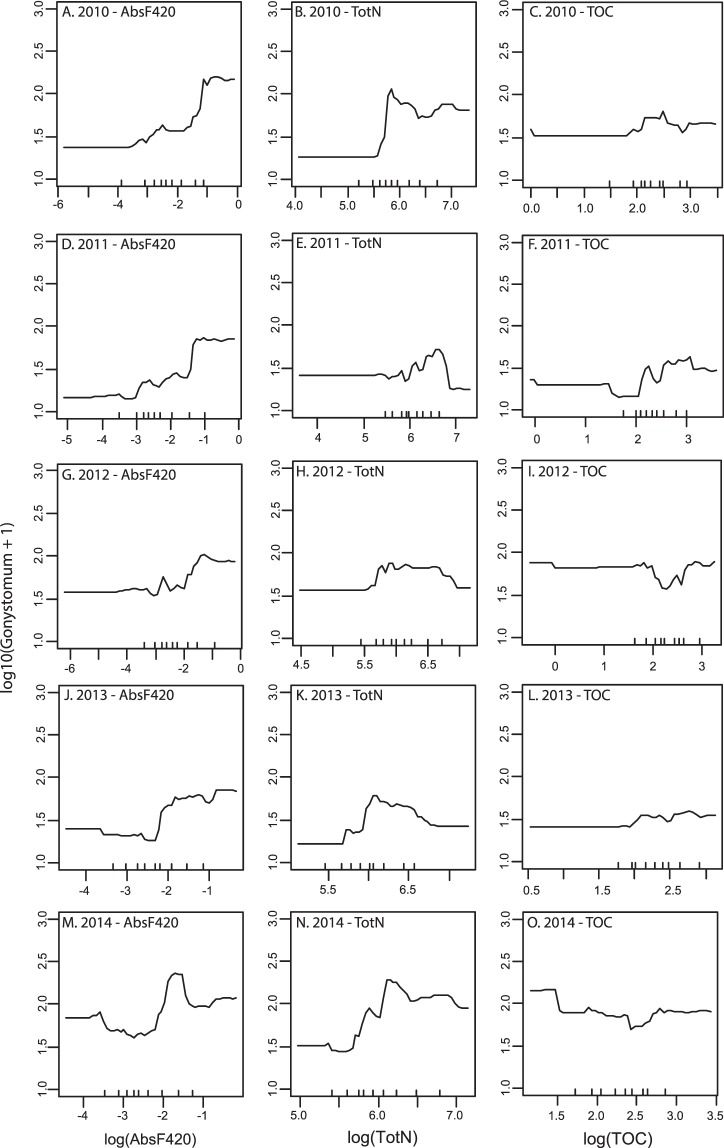
Partial dependency plots between *G*. *semen* abundances and absorbance at 420 nm (**A**,**D**,**G**,**J** and **M**) concentration of total nitrogen (**B**,**E**,**H**,**K** and **N**) and concentration of TOC (**C**,**F**,**I**,**L** and **O**) for the years 2010 to 2014. A linear relationship would show up as a straight line.

When analyzing the marginal linear influence of iron on *G*. *semen* abundances relative to the variables with VIP > 1 with stepwise regression, iron concentration only had a marginal contribution one year (2010, Table 2). Phosphorus concentrations and pH explained most marginal variation (together around 30%) in *G*. *semen* abundance in linear models, while absorbance, TOC and total nitrogen concentrations were unimportant. An explanation for the discrepancy between the PLS and the stepwise regressions may be that relationships are non-linear. Dependency plots confirmed that the explanatory variables tested showed a non-linear relationship with *G*. *semen* abundance (Figs 2 and 3). However, for TOC the dependency plot show that the concentration was not correlated to *G*. *semen* abundance (Fig. 3). The dependency plots further suggested iron, pH and total phosphorus concentration to have the highest independent impact on *G*. *semen* abundance as these variables spanned the largest range in *G*. *semen* abundance (Figs 2 and 3).

**Table 2:** Partial r^2^ –values for the explanatory variables with VIP > 1 from stepwise linear regression on abundance of *G*. *semen* each year.

Year	AbsF420	Fe	pH	TOC	TotN	TotP
2010	Not Incl.	0.03	0.06	Not Incl.	Not Incl.	0.06
2011	Not Incl.	Not Incl.	0.15	Not Incl.	Not Incl.	0.09
2012	Not Incl.	Not Incl.	0.22	Not Incl.	Not Incl.	0.21
2013	Not Incl.	Not Incl.	0.29	Not Incl.	Not Incl.	0.13
2014	Not Incl.	Not Incl.	0.21	Not Incl.	Not Incl.	0.16

‘Not Incl.’ means a variable was not included in the final model based on model AIC.

**Figure 4:**
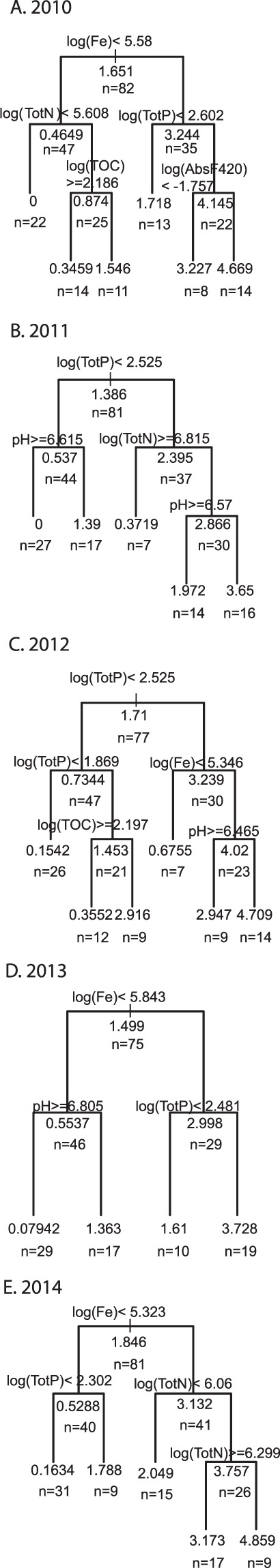
Regression tree for the years 2010 to 2014 explaining *G. semen* abundances using the most important variables according to the PLS analysis.

This result was further confirmed by the regression trees which showed that high iron and phosphorus concentrations in conjunction with low pH seem to result in high *G*. *semen* abundance, in a non-linear way (Fig. 4). In all years studied at least one of these variables and up to all three (2012) were included as nodes associated with the highest abundance of *G*. *semen* (the rightmost path).

From these analyses it is not possible to identify the single most important variable associated with high *G*. *semen* abundance. Instead they show several variables acting in coherence, where the ideal *G*. *semen* lake appear to be an acidic one with phosphorus and iron concentrations above 15 μg l^−1^ and 200 μg l^−1^, respectively (Supplementary Fig. S2). Interestingly, the lakes from agricultural areas with highest phosphorus concentration lack *G*. *semen* completely likely because of high pH and low iron concentration (data not shown). *G*. *semen* has been shown to be highly plastic regarding pH, and being able to grow between pH 5 and pH 8^9^ which is approximately the range of pH observed in our lakes. Still, our results are in agreement with previous studies showing that lower pH is favoring high abundances of *G*. *semen*^2,10^. However, it should be noted that, according to our data, the ideal *G*. *semen* lake is in no way extreme (Supplementary Fig. S2), but has a pH slightly lower than 7. An association between *G*. *semen*, and phosphorus has also been observed in previous studies^1,2,5^. However, the “threshold value” in phosphorus concentrations between lakes with and without *G*. *semen* observed here (Supplementary Fig. S2) is also not extreme, but corresponds approximately to oligo-mesotrophic conditions. Still, according to our data *G*. *semen* blooms are not expected in very oligotrophic lakes.

Since the results regarding pH and phosphorus were more or less expected the novelty of our study is that iron concentration seems to have a larger impact on *G*. *semen* abundance than TOC or absorbance *per se* which previously have been considered to be important steering factors for *G*. *semen* abundance^2,10^. This conclusion is based on our regression tree analysis, where water color (AbsF420) and TOC concentration appeared to be of relatively low importance for determining the abundance of *G*. *semen* (Fig. 4). Thus, *G*. *semen* blooms are associated with brown waters but not because of carbon but rather due to iron. The question arising from this result is then: What may be the reasons for high iron concentrations leading to blooms of *G*. *semen*?

Iron limitation of primary production in the ocean is well-documented^43,44^. In lakes, iron limitation of primary production has been demonstrated in, for instance, oligotrophic clear-water lakes^43^ and references therein^45^. Several authors have also reported that increased iron concentrations may promote cyanobacteria blooms especially because iron is essential for nitrogen fixation^46,47^. To our knowledge, however, no study previously reported a connection between iron concentrations and abundance or occurrence of *G*. *semen* in lakes. Iron is, in general, a key micronutrient for phytoplankton since it is essential for some metabolic processes such as photosynthesis and respiration^43^ and references therein^44^. *Gonyostomum semen* might have high requirements for iron as it has a large number of chloroplasts, which, in turn, may be an effect of *G*. *semen* being adapted to the low light environment of dark water color lakes^2,5,10^. Phytoplankton cells can adapt to low light environment by increasing the number of photosystem I (PSI) unit per cells in order to capture more photons, however, the PSI is a sink for iron as it requires 12 atoms of iron per PSI units^43^. Thus, iron is likely an important resource for this kind of adaption in phytoplankton cells.

Several authors have reported darker water color not only in Swedish lakes but also elsewhere in the boreal zone^13,14^, and iron has recently been shown to be an important driver of this increase in northern latitude lakes^13^. Observed ecosystem effects of browning are for instance increases in lake net heteroptrophy due to light limitation and increased supply of organic substrates^48^. In general, studies of browning effects had, so far, a strong focus on TOC or light limitation effects on ecosystems rather than iron^49^. The ecosystem effects of *G*. *semen* growth has only received little attention to date, but current knowledge indicates that since *G*. *semen* is a poor food source for grazers, and, thus, heterotrophic food chains are favored over photosynthesis based food chains as grazers shift their diet toward more bacteria which is more easily to graze^7^. Alternatively, it is possible, but to our knowledge not yet investigated, that *G*. *semen* blooms may decrease the degree of net heterotrophy of lakes due to high photosynthesis rates. This pattern would represent a departure from current expected effects following water browning (i.e., increased net heterotrophy), and showcase an unexpected impact of an invasive species on ecosystem functioning.

We should note that the results reported here are based purely on correlations and should not be seen as a proof of an effect of iron on *G*. *semen* abundances. For instance, it is possible that some important unmeasured factors for *G*. *semen* abundance were strongly correlated to iron concentrations in the lakes and, thus, lead to spurious correlations with iron. Still, given the large number of parameters included in our model, we believe that iron may play an important role in the increase in *G*. *semen* abundance in boreal lakes alongside other known parameters such as total phosphorus concentration and pH. Here we identify lakes with iron concentrations >200 µg l^−1^, pH < 7 and phosphorus concentrations >15 µg l^−1^ to have a large probability of having *G*. *semen* blooms. We suggest that the results presented here should be used as a starting point for experimental studies investigating the role of iron for *G*. *semen* growth and subsequent ecosystem effects.

## Electronic supplementary material

Supplementary Information

## Data Availability

All the data obtained from the Swedish national lake survey are available on the website: http://miljodata.slu.se/mvm/. The absorbance and fluorescence spectra are available on the online repository at: http://urn.kb.se/resolve?urn=urn:nbn:se:uu:diva-341683.
