# Supplementary material for: High abundances of the nuisance raphidophyte Gonyostomum semen in brown water lakes are associated with high concentrations of iron
Source: Sci Rep. 2018 Sep 7;8:13463. doi: 10.1038/s41598-018-31892-7 (PMC6128840; doi:10.1038/s41598-018-31892-7)

High abundances of the nuisance raphidophyte *Gonyostomum semen* in brown water lakes are associated with high concentrations of iron

Karen Lebret[1,2*], Örjan Östman[3], Silke Langenheder[1], Stina Drakare[4], François Guillemette[5], Eva S. Lindström[1]

[1] Department of Ecology and Genetics/Limnology, Uppsala University, Norbyvägen 18D, SE-752 36 Uppsala, Sweden

[2] Centre for Ecology and Evolution in Microbial model Systems - EEMiS, Department of Biology and Environmental Science, Linnæus University, SE-391 82 Kalmar, Sweden.

[3] Department of Aquatic Resources, Swedish University of Agricultural Sciences, Skolgatan 6, SE-742 42, Öregrund, Sweden.

[4] Department of Aquatic Sciences and Assessment, Swedish University of Agricultural Sciences - SLU, PO Box 7050, SE-750 07 Uppsala, Sweden

[5] Research Center on Watershed – Aquatic Ecosystem Interactions (RIVE), Department of Environmental Sciences, Université du Québec à Trois-Rivières, Québec, Canada

Table S1: Summary of results from PLS analysis showing the explained variance of the variables and *G. semen* abundance and the predictive power of the PLS model for *G. semen* abundance for each year.

Year (number of lakes)	R^2X (explained variance of the variables)	R^2Y, (explained variance of *G.semen* abundance)	Q^2 (Predictive power of the training model)
2010 (90)	0.24	0.39	0.34
2011 (90)	0.28	0.23	0.19
2012 (91)	0.32	0.29	0.26
2013 (93)	0.22	0.38	0.34
2014 (95)	0.30	0.33	0.30
2014 with EEMs (72)	0.23	0.38	0.28

Figure S1: Loading plots of the two first components from the PLS analyses of *G. semen* abundances for 2010 (A), 2011 (B), 2012 (C), 2013 (D), 2014 without the EEMs variables (E) and 2014 including the EEMs and PARAFRAC variables (F).

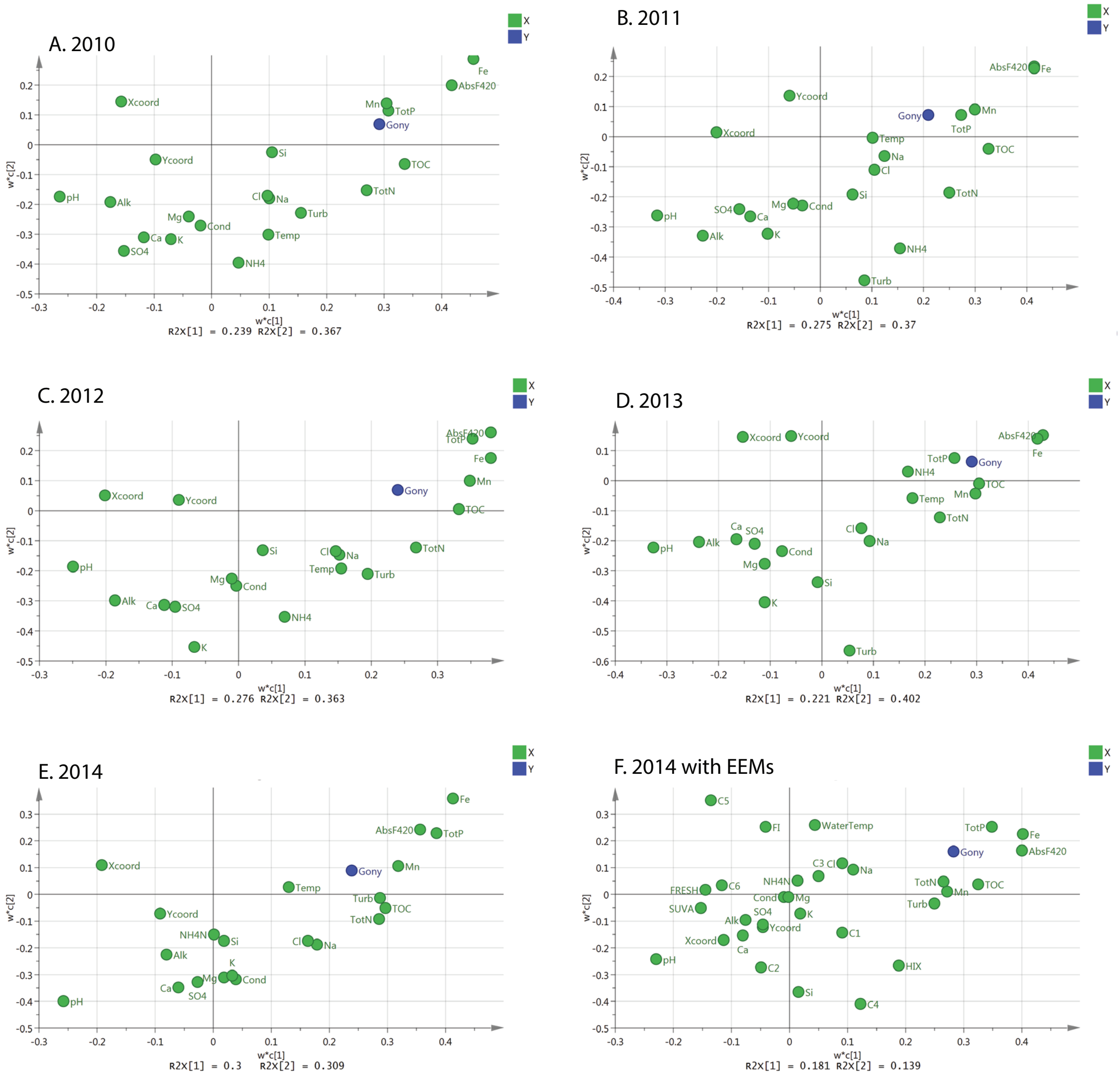

Figure S2: Boxplot of iron concentrations (A), pH (B) and total phosphorus concentrations (C) with and without *G. semen* in the 95 lakes from 2010 to 2014. Note that Fe and TotP values are presented in log scale. The boxes represent 50 % of the data and the whiskers an additional 25% of the data.

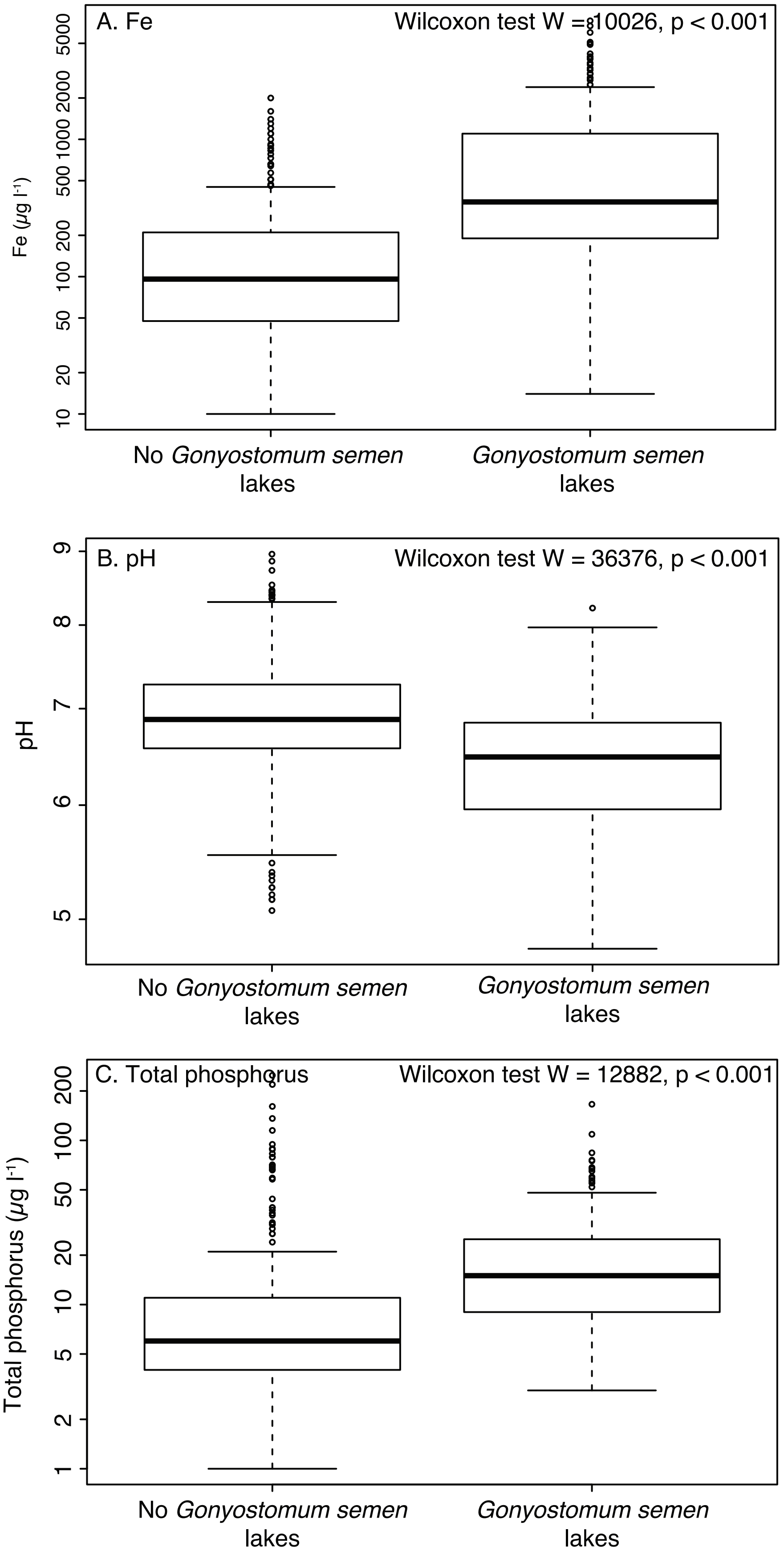

Supplement: Supplementary file 1 — Supplementary Information [file 41598_2018_31892_MOESM1_ESM.pdf]
